# Pharmacological Properties to Pharmacological Insight of Sesamin in Breast Cancer Treatment: A Literature-Based Review Study

**DOI:** 10.1155/2022/2599689

**Published:** 2022-02-17

**Authors:** Md Sohel, Md. Nurul Islam, Md. Arju Hossain, Tayeba Sultana, Amit Dutta, Md. Sohanur Rahman, Suraiya Aktar, Khairul Islam, Abdullah Al Mamun

**Affiliations:** ^1^Department of Biochemistry and Molecular Biology, Mawlana Bhashani Science and Technology University, Santosh, Tangail 1902, Bangladesh; ^2^Department of Pharmacy, Mawlana Bhashani Science and Technology University, Santosh, Tangail 1902, Bangladesh; ^3^Department of Biotechnology and Genetic Engineering, Mawlana Bhashani Science and Technology University, Santosh, Tangail 1902, Bangladesh; ^4^Department of Biochemistry and Molecular Biology, Trust University, Barishal, Ruiya, Nobogram Road, Barishal 8200, Bangladesh; ^5^Department of Biochemistry and Molecular Biology, Rajshahi University, Bangladesh

## Abstract

The use of dietary phytochemical rather than conventional therapies to treat numerous cancers is now a well-known approach in medical science. Easily available and less toxic dietary phytochemicals present in plants should be introduced in the list of phytochemical-based treatment areas. Sesamin, a natural phytochemical, may be a promising chemopreventive agent aiming to manage breast cancer. In this study, we discussed the pharmacological properties of sesamin that determine its therapeutics opportunity to be used in breast cancer treatment and other diseases. Sesamin is available in medicinal plants, especially in *Sesamum indicum*, and is easily metabolized by the liver. To better understand the antibreast cancer consequence of sesamin, we postulate some putative pathways related to the antibreast cancer mechanism: (1) regulation of estrogen receptor (ER-*α* and ER-*β*) activities, (2) suppressing programmed death-ligand 1 (PD-L1) overexpression, (3) growth factor receptor inhibition, and (4) some tyrosine kinase pathways. Targeting these pathways, sesamin can modulate cell proliferation, cell cycle arrest, cell growth and viability, metastasis, angiogenesis, apoptosis, and oncogene inactivation in various *in vitro* and animal models. Although the actual tumor intrinsic signaling mechanism targeted by sesamin in cancer treatment is still unknown, this review summarized that this phytoestrogen suppressed NF-*κ*B, STAT, MAPK, and PIK/AKT signaling pathways and activated some tumor suppressor protein in numerous breast cancer models. Cotreatment with *γ*-tocotrienol, conventional drugs, and several drug carriers systems increased the anticancer potentiality of sesamin. Furthermore, sesamin exhibited promising pharmacokinetics properties with less toxicity in the bodies. Overall, the shreds of evidence highlight that sesamin can be a potent candidate to design drugs against breast cancer. So, like other phytochemicals, sesamin can be consumed for better therapeutic advantages due to having the ability to target a plethora of molecular pathways until clinically trialed standard drugs are not available in pharma markets.

## 1. Introduction

Several diseases, from infectious to noninfectious, have become a burden globally. Cancer, especially breast cancer, is a noninfectious public health-related disease, considered the 2^nd^ second most frequent cancer type after lung [1]. According to the literature review, conventional treatment modalities are surgery, radiation therapy, chemotherapeutics drugs, hormone therapy, targeted therapy, and immune therapy [2]. But these types of therapies are highly specific and are not effective against all types of breast cancer. For example, chemotherapy is the only fruitful and effective therapy against estrogen nonresponsive cancer [3]. Furthermore, some breast cancer treatment strategies cause significant side effects that continue or appear months or after treatment has already ended [4]. Some chemotherapeutic drugs are becoming resistant to cancer cells [5]. So we should not be limited to these conventional therapies for breast cancer management. The use of natural-based phytochemicals should be given priority against both infectious [6] and noninfectious diseases [7, 8]. Hormone, i.e., estrogen, plays a significant role in breast cancer development [9] by stimulating breast cancer growth and promoting the normal and the neoplastic breast epithelium [10]. Phytochemicals can modulate estrogen receptors and several other growth factors. In this regard, phytoestrogen is the potent candidate due to its structural similarity with estrogen receptors [11, 12]. Phytoestrogens are reported to accelerate breast cancer cell growth at a lower concentration, but at higher concentrations, they may suppress cancer progression by controlling some regulatory point through ER-*α*, ER-*β* [13], and HER2 [14] and aromatase controlling [15].

Sesamin is a water-insoluble phytoestrogen, categorized into lignans, and recently received considerable attention from scientists that could be used to treat breast cancer. *Sesamum indicum* is a major source of sesamin, but other 30 medicinal plants in some specific genera contain minor amounts of sesamin [16]. Sesamin is widely used against inflammatory diseases [17], neurodegenerative disease [18], liver disease [19], diabetes, eye problem [20], cardiovascular disease [21], and lung disease [22]. Furthermore, the anticancer activities of sesamin have been documented against numerous human cancers in various *in vitro* and animal models [23]. However, therapeutic advantage of sesamin again breast cancer is limited. Therefore, we aimed to make an overview of sesamin and breast cancer, which will open a new door in the near future. The major advantages of using sesamin in breast cancer treatment are its availability in nature, structural chemistry, and formation of nanocarrier to increase bioavailability. Furthermore, sesamin possesses promising drug-likeness properties, synergistic activities, less toxicity, and its metabolic intermediate. Metabolites of sesamin also possess anticancer activities against breast cancer [24, 25]. Dietary consumption of sesamin is attributed to anticancer activities by regulating cell death and viability by regulating cell cycle and proliferation, apoptosis-related signal transduction, angiogenesis and metastasis pathway, hypoxia regulation, and other unknown investigating the mechanism in *in vitro and in vivo* model systems.

This review summarizes about sesamin from sources, structure, metabolism, molecular pharmacology with anticancer mechanisms, and pharmacokinetics with a future prediction.

## 2. Overview of Sesamin Distribution

Sesamin is a type of lignan found in vascular plants [26]. Sesame species (*Sesamum indicum* L.) is one of the major sources of this sesamin [27], and Gomazou, a Japanese variety of sesame seeds, is reported to contain sesamin about 15 mg/1 gm dry weight of its seed [28]. The oil extracted from the sesame seed also contains sesamin that can be used for cooking and cosmetic preparation [29]. Followed by seed, leaves have a small amount (2.6 g g^−1^ dry weight) of sesamin detected by Ultra Performance Liquid Chromatography-Fluorescence Detection (UPLC-FLD) [28]. Other than sesame seed, sesamin has been identified from stems and roots of several medicinal plants species, including *Piper* genus [30], *Magnolia coco*, *Bridelia retusa*, *Phyllarthron comorense*, *Zanthoxylum tetraspermum*, and *Stemona collinsae* [31–36]. Chinese wild ginger, paulownia, and gingko were also reported to contain sesamin. Sesamin was also detected as an endogenous secondary metabolite of a parasitic plant like *Cuscuta palaestina.* Some reported sources of sesamin are listed in Table 1.

### 2.1. Chemistry

**Figure 1:**
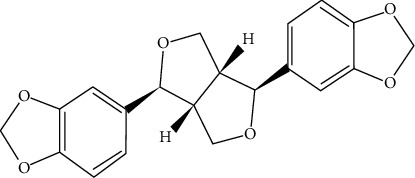
Chemical structure of sesamin.

Sesamin was first isolated in 1890 from the sesame seeds. It belongs to the benzodioxole family. The IUPAC name of this lignans is (1S,3a R,4 S,6a R)-1,4-bis (benzo[d][1,3]dioxol-5-yl)tetrahydro-1 H,3H-furo [3,4-c] fura, and the chemical formula of sesamin is C_20_H_18_O_6_ [46]. It contains 26 heavy atom counts, 1 covalently bonded unit amount, and no formal charge [46]. The molecular weight of sesamin is 354.35 g/mol, but the exact mass is 354.36 [26, 47]. This chemical compound contains two fused dihydrofuran with two benzene rings replaced with a group of methylene dioxide (-O-CH2-O-) at the 3 and 4 symmetrically attached to each of the carbon atoms near to the ether oxygen atoms [48]. Furthermore, this phytoestrogen does not contain any nonconjugated amine and carboxylic acid groups.  Figure 1 portrays the chemical structure of sesamin.

### 2.2. Metabolism

Metabolism of sesamin appears to be species-specific. Liu summarized that sesamin was metabolized to other substances in the gut or liver [49]. The primary metabolic products of sesamin are enterodiol (ED) and enterolactone (EL), produced through fermentation with human fecal microbiota and colonic microflora [50]. Sesamin is successively metabolized in the liver by liver microsomes. Hydroxylated metabolites of sesamin were released in the bile in the liver [51], where cytochrome P450 is an essential enzyme in the liver [52]. According to the previous report, sesamin was converted into monocatechol metabolite (SC-1), and it was metabolized into glucuronide of SC-1 (SC-1-GlcUA) and methylated metabolites of SC-1 (SC-1m) by DP-glucuronosyltransferase and catechol O-methyl transferase, respectively, in the liver [53]. Recently, Sakaki added that CYP2C9 is the predominant enzyme for sesamin metabolism in the human liver [54]. Recently, in the case of microorganisms, i.e., *Sinomonas* spp. no. 22, enzyme SesA converts sesamin to their respective intermediate [55].

## 3. Sesamin with Nanoformulation to Increase the Bioavailability

Sesamin is fat-soluble or poorly water-soluble lignans (2.5 *μ*g/ml), significantly limiting its dissolution rates and release efficiency [56]. Improving the absorption and distribution of lipophilic components like sesamin can be possible by dissolving oil or various nanocarriers, i.e., a self-nanoemulsifying drug delivery system (SNEDD). Wang summarized that after self-emulsification of SNEDDS, droplet size was dispersed by sesamin at 66.4 ± 31.4 nm with increased intestinal permeability, relative bioavailability, and absolute bioavailability by more than three-fold and 12.9-fold and 0.3% to 4.4%, respectively [57]. Iwamoto et al. stated that mixing with turmeric oil could increase the bioavailability of sesamin [58], where sesame extract with a turmeric oil mixture of sesame extract and turmeric oil (MST) was fed to Slc:ddY mice, and found that serum sesamin contents in the MST-treated group were 23-fold higher compared to the control group (administering sesame extract alone). Sato et al. state that solid dispersion (SD) approach increased solubilizing effect of sesamin by using *α*-glycosylated stevia (Stevia-G) carrier compounds [56]. They found that sesamin-loaded SD with Stevia-G (sesamin/Stevia-G-SD) (20 mg sesamin) increased bioavailability around 30-fold higher with 190-fold dissolution than that of crystalline sesamin in Sprague–Dawley rats (200 ± 50 g, 6–9 weeks). Furthermore, an experimental study by Ebrahimi summarized that formulation containing sesame oil (a significant source of sesamin) reduced the average droplet size of microemulsion samples with a range of 16.6 ± 0.1-64.6 ± 0.2 nm with a polydispersity index (PDI) value of less than 0.5 [59]. So, the formulation procedure of sesamin has excellent potential for oral administration, enhancing its pharmacological application value.

## 4. Molecular Pharmacology of Sesamin in Breast Tissue

**Figure 2:**
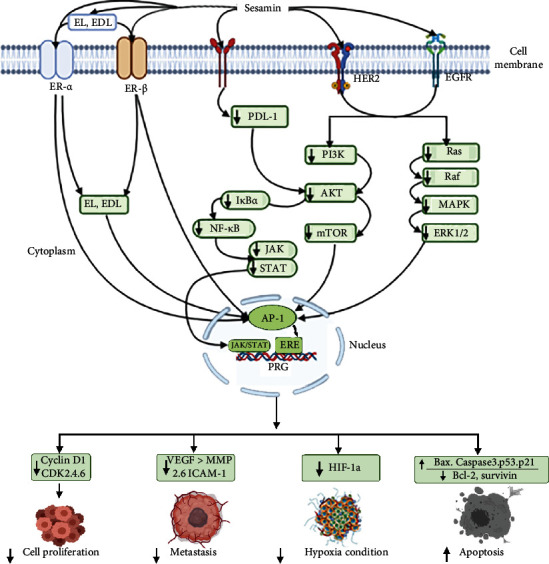
Graphical presentation of the molecular pharmacology of sesamin in breast cancer. Sesamin and its metabolites have potential antibreast cancer activities. In the early stage, the mechanism is mediated through regulation of extracellular receptor-like estrogen receptor-*α* (ER-*α*), estrogen receptor-*β* (ER-*β*), programmed death-ligand 1 (PD-L1), Herceptin epidermal growth receptor 2 (HER-2), and epidermal growth factor receptor (EGFR). Through this receptor, sesamin and its metabolites inhibit some major signaling molecules like pAkt-PI3K/mTOR and JAK/STAT. These tumor intrinsic signals upregulate activator protein 1 (AP-1), inhibit estrogen-responsive elements (ERE), and activate other regulatory proteins. Sesamin and metabolites finally inhibit cell cycle progression by the suppression of cyclin and its associated enzyme kinase (CKD 2, 4, and 6); inhibit metastasis and angiogenesis by the suppression of MMP 2, MMP 9, ICAM-1, and VEGF; and induce apoptosis by the upregulation of Bax, caspase 3, P^21^, and P^53^ with downregulation of Bcl2 and survivin protein.

Sesamin is a type of phytoestrogen that belongs to lignans [60], associated with several pharmacological activities against breast cancer through regulating several receptors, like estrogen receptor-*α* (ER-*α*), receptor-*β* (ER-*β*), G protein-mediated signaling pathways, growth factor receptor, i.e., HER2 and EGFR, and some receptor tyrosine kinase (RTK). Estrogen receptor-*α* plays a significant role in estrogen-responsive breast cancer initiation and progression [61, 62]. Sesamin competitively bound with estrogen receptor in the form of antagonist and showed inhibitory activities on estrogen-mediated estrogen-responsive element (ERE) induction in T47D-KBluc cells at 10^−9^-10^−6^ M [63]. There is huge of evidence that claims receptor tyrosine kinase (RTK): for instance, HER2 and EGFR [64], IGFIR [65], and hepatocyte growth factor receptor (HGFR) [66] are tumor initiators in breast tissue. So targeting these receptors is a novel strategy to treat breast cancer. Truan et al. summarized that sesamin suppressed the expression of HER2 and EGFR receptors and their activities in MCF-7 cancer cell lines and athymic mice [67]. It is found in some subtypes of metastatic breast cancer, particularly triple-negative breast cancer, the protein programmed death-ligand 1(PD-L1) expressed highly, so targeting PD-L1in breast cancer is a potential complementary therapy in cancer patients [68]. Kongtawelert et al. summarized that sesamin (200 *μ*M) suppressed PD-L1 expression through AKT, NF, and JAK/STAT signaling inhibition in MDA-MB 231 breast cancer cell lines [69]. COX-2 enzyme mediates CYP-19 transcription and aromatase, which caused an increase in biosynthesis of estrogen and estrogen-responsive breast cancer [70]. However, information about sesamin-COX-2-related breast cancer treatment is rare, but sesamin could decrease COX-2 expression by inducing apoptosis and G1 phase arrest by targeting pAkt-PI3K signaling in lung cancer [71]. However, the interaction of sesamin with the vascular endothelial growth factor receptor-2 (VEGFR-2) and insulin-like growth factor-1 (IGF-1R) signaling pathway in breast cancer cells is yet not established. Moreover, some of the sesamin metabolic intermediates have strong interaction with estrogen receptors and mediate anticancer activity. For instance, enterolignans, i.e., enterolactone and enterodiol, impede human estrogen receptor (ER) signaling in hormone-dependent breast cancer. These two enterolignans modulate ER-*α* and ER-*β* at the transcriptional level by regulating ER-*α*-targeted genetic elements by transactivation functions AF-1 and AF-2 [72]. The molecular pharmacology of sesamin in breast cancer is summarized in Figure 2.

## 5. Anticancer Effects of Sesamin and Their Metabolites

### 5.1. *In Vitro* Study of Sesamin

Sesamin has been shown to exhibit *in vitro* anticancer activities against various breast cancer cell lines. Kongtawelert et al. conducted the antibreast cancer activities of sesamin and reported that sesamin (0-200 *μ*M) inhibited cell proliferation with suppressing metastasis by the inactivation of oncosignaling pathways like P13K/AKT, NF-*κ*B, ERK, and JAK/STAT in MDA-MB 321 cell lines [69]. Furthermore, this phytochemical also arrested the G1 phase and downregulated PDL-1, MMP-9, and MMP-2 resulted in inhibition of cell migration at the same dose and cell line. Yokota et al. manifested the cell proliferation capability of sesamin in the breast cancer cell and found that sesamin inhibited cell proliferation through decreasing cyclin D1 gene expression that mediates cyclin D1 degradation with concomitant increasing retinoblastoma protein dephosphorylating, leading to growth inhibition at the dose of 1-100 *μ*M [73]. In addition, sesamin (51.1 *μ*M) inhibits cell proliferation through the mitochondrial-mediated pathway by downregulating Bcl-2 and cyclin D1 expression [74]. However, sesamin (25-100 *μ*M) halted cancer progression by inhibiting the NF-*κ*B, STAT3, JNK, ERK1/2, MAPK, and PI3K/AKT and activating tumor suppressor protein like p38 and p53 via downregulating tumor necrosis factor-alpha (TNF-*α*) [74, 75]. Sesamin (98.57 *μ*M) decreased the cell viability and cytotoxic effect in MCF-7 breast cancer [76]. Similarly, sesamin (50 *μ*M) declines cell viability with inducing apoptosis through the underline mechanism of activating Bax, caspase 3, and tumor suppressor protein p53, leading to G1 phase cell cycle arrest [77]. Macrophage-induced proangiogenic activities were inhibited by sesamin (50-100 *μ*M) in breast cancer cell line through the underlying mechanism of inhibiting major transcription factors including HIF-1*α* and NF-*κ*B and signaling mechanism of ERK, JNK, and PI3K and metastasis factors including VEGF and MMP-9 in MCF-7 and MDA-MB 231 cancer cell [78]. Additionally, sesamin (50 *μ*M) can decrease cell growth and cell viability by increasing apoptosis through the chain transfer pathway by upregulating pS2 and progesterone receptor genes in T-47D breast cancer cells [63].

### 5.2. Anticancer Activity of Sesamin Metabolites (Enterolactone and Enterodiol)

Sesamin is a plant compound, metabolized by the liver to produce some estrogenic metabolic intermediate. These metabolites link with estrogen receptors, but their mechanism of action, either estrogenic or antiestrogenic, is still unknown. Enterolactone and enterodiol are initial metabolites produced in a minor amount [50]. Liu et al. summarized that both metabolites have antibreast cancer activities, but enterolactone attributes more potent anticancer activity with fewer side effects than enterodiol [79].

Enterolactone (25-75 *μ*M) was found to downregulate MMP-2 and MMP-9 matrix enzyme expression with upregulating their inhibitors including TIMP-1 and TIMP-2. These are significant inhibitors of MMP-2 and MMP-9, resulting in the regulation of migration of breast cancer cells during metastasis in cell line MDA-MB 231 [24]. Furthermore, MDA-MB 231cell growth inhibition of enterolactone was evident by accumulating cells at the S phase through the underlying mechanism of lowering cell cycle regulatory proteins cyclin A2, cyclin B1, and cyclin E1 genes expression without changing CDK4, CDK6, and cyclin D1 for G0/G1 phase regulation [80]. Enterolactone (10 ng/ml) also mediated the anticancer mechanism by interfering with TGF-*β*-induced EMT through blocking the ERK/NF-*κ*B/snail, MAPK-p38, and a cluster of differentiation 44 (CD44) with upregulating the epithelial markers E-cadherin and occluding in similar cell lines [81]. Like enterolactone, enterodiol possesses anticancer activity by modulating cell migration and proliferation. Carreau et al. found that enterodiol suppressed MMP-2-9 and regulated MMP secretion in estrogen-responsive breast cancer cell MCF-7 [72]. However, the anticancer activity of other sesamin's metabolites is still unknown. Summary of sesamin activities in breast cancer are listed in Table 2.

### 5.3. *In Vivo* and Clinical Trial Study

Sesamin has been widely tested in preclinical and clinical trials for several diseases, i.e., ischemic brain stroke [83], depression [84], Parkinson's disease [85], osteoarthritis [86], diabetic retinopathy [20], and acute hepatic injury [87], but therapeutic activities of sesamin in *in vivo* breast cancer model are limited. Sesamin (1 g/kg, 8 wk the basal diet) supplementation in athymic mice reduced tumor size by around 23% compared to control through downregulating growth factor receptor including EGFR and HER2 and reducing pMAPK expression [67]. Furthermore, administration of DMBA for weeks in female Sprague-Dawley decreased the cumulative number of palpable mammary cancer by 36% in rats on a control diet. Again, sesamin reduced fatty acid in plasma and liver, and tumor phosphatidylcholine decreases the serum prostaglandin E2 in the sesamin group [88]. In a randomized, placebo-controlled, crossover study conducted by Wu et al., with 26 healthy postmenopausal women, they found that sesamin (50 gm, five weeks) increased antioxidant status [89]. Although there is limited information on *in vivo* and clinical trials due to the lack of study, conducting more studies may reveal sesamin as the potential anticancer agent.

### 5.4. The Synergetic Activity of Sesamin in Breast Cancer Treatment

Synergy occurs when two or more substances work combined to produce a more considerable effect than the sum of their individual effects [75]. Information about the synergistic activity of sesamin in breast cancer treatments is scarce. Initially, Akl reported that sesamin (10-120 *μ*M) with *γ*-tocotrienol synergistically inhibits growth-mediated EGF-dependent by decreasing phosphorylation of ErbB3 and ErbB4 receptor and mitogenic signaling, i.e., suppressed intracellular and phosphorylated oncogene c-Raf, MAPK/ERK kinase, extracellular signal-regulated kinase 1/2, phosphoinositide-dependent kinase-1, phosphoinositide-3-kinase, protein kinase B, p-NF-*κ*B, JAK1, JAK 2, and STAT1 at a dose-dependent manner in mammary tumor cells [91]. Furthermore, one year later, they found that the antiproliferation actions of *γ*-tocotrienol synergize sesamin in neoplastic mouse (+SA) and estrogen-responsive and nonresponsive (MCF-7 and MDA-MB 231) breast cancer cell line. These synergistic activities are mediated by regulating cell cycle regulatory protein, i.e., activating retinoblastoma protein (p-RB, decreasing), cyclin D1, and its associated enzymes CDK2, CDK4, CDK6, and E2 transcription level 1, and upregulating tumor suppressor proteins p27 and p16 mediates the arrest cell cycle at the G1 phase [92]. As a result, to prevent or treat breast cancer, the synergistic growth inhibitory effects of *γ*-tocotrienol and sesamin treatment can be leveraged as a viable anticancer therapeutic strategy.

## 6. Toxicological Potential of Sesamin

Plant lignans, including sesamin, the most frequent lignans in sesame seed, are always beneficial to health [93]. Sesamin has many pharmacological advantages and can be used for hyperlipidemia, hypertension, and cancer treatment [75]. However, it has few toxicities toward normal cells [91]. Sesame seed oil decreases blood pressure, so sugar levels should be monitored while taking sesame seed oil [94]. Several studies show that sesamin might cause an allergic reaction in some people. Sesame oil can be used as a nasal spray, but it causes nasal dripping and blockage. People who already have low blood pressure and diabetes taking excessive sesame might lower blood sugar levels and drop blood pressure. Sesame also may interfere with blood sugar levels during or after surgery [95]. In microorganisms and animals, sesamin (155 *μ*M) specifically inhibiting delta-5 desaturase decreased polyunsaturated fatty acid biosynthesis [96]. Sesamin is familiar with positively affecting HMG-CoA reductase [95] and lipid metabolism or fatty acid oxidation-related enzymes [96] by modulating their mRNA levels. Yasuda et al. summarized that sesamin significantly inhibits CYP2C9, CYP21A2, and CYP23A4 in a dose-dependent manner [52], leading to drug toxicity, drug-drug interactions, and other adverse effects. Sesamin also inhibited the tocopherol [97] and arachidonic acid metabolism [97] by inhibiting CYP4F2. Therefore, beside the pharmacological advantages, it is important to consider the toxic effect of any naturally derived phytochemicals before use. However, more studies can help to reduce the toxicity or determine the effective safe dose of sesamin for the treatment of diseases.

## 7. Pharmacokinetics Prediction of Sesamin

In current drug discovery efforts, small molecule leads with appealing pharmacokinetic characteristics are typically sought out. So we liked to determine the pharmacokinetics properties of sesamin. ADME/Tox (absorption, distribution, metabolism, elimination, and toxicity) profile of sesamin was predicted using online accessible Swiss ADME [98](drug-likeness properties), pkCSM (absorption, distribution, excretion, and toxicity) [99], and admet SAR [100] (metabolism) in *in silico* tools and listed in Table 3.

Our predicted result demonstrated that sesamin was within an acceptable range in all tested Lipinski, Ghose, Egan, Veber, and Muegge rules with a good bioavailability score (0.55), indicating sesamin maintained drug-likeness properties. Absorption is an essential property in drug discovery. Our predicted result showed that sesamin is highly soluble in water (-4.223), highly absorbed by the human intestine (97.81%), highly permeable to the skin, and Caco-2 with limited glycoprotein status. Distribution is followed by absorption, another principal descriptor for drug development, which depends on the aqueous solubility of compounds. Our *in silico* result revealed that sesamin could penetrate the central nervous system (CNS) and blood-brain barrier(BBB) with a poor steady-state volume of distribution (VDss). Human cytochrome P450 (CYP) isoforms are involved in drug metabolism in the liver, and its inhibition causes drug toxicity in the body. The predicted metabolic result reported that sesamin has poor metabolic status in both CYP isoform substrate and inhibition. Excretion property based on the total renal clearance parameter was predicted, where the sesamin's total clearance (logCLtot) was -0.126 ml/min/kg. The toxicity profile of sesamin has been predicted based on eye corrosion, hepatotoxicity, AMES toxicity, and hERG potassium channel inhibition. The results outlined that sesamin showed toxicity only in the AMES test rather than eye corrosion, hepatotoxicity, hERG potassium channel inhibition, and *T. pyriformis*. P-glycoprotein (ATP-binding cassette (ABC) inhibition, aromatase, and estrogen receptor targeting of this protein) is a potent mechanism for any type of cancer treatment. Our analysis revealed that sesamin is a potent inhibitor of P-glycoprotein and can modulate estrogen receptors, but it does not inhibit aromatase enzymes.

To sum up, in our study, sesamin attributes some pharmacokinetics profiles (Table 3). So sesamin will facilitate the drug discovery process in computational chemistry, i.e., docking analysis, neural networking study, and pharmacophore-based virtual screening campaigns for the drug discovery community.

## 8. Limitation and Future Prospect of Sesamin in Breast Cancer Biology

The traditional treatment approaches used for breast cancer treatment are interrupted by many factors. Sesamin has a broad range of pharmacological properties that could be useful in therapeutics practices. Some of these properties contribute to sesamin as a potent anticancer agent. However, beside the pharmacological advantages, there is a major limitation to anticipate the real potentiality of sesamin against breast cancer treatment due to limited research on it. So, before delivering this phytochemical to the medicine cabinet as a natural therapeutic agent or medicament, it must first be approved by testing *in vitro*, *in vivo*, and clinical trial (stages I-IV). Long research is still needed to find out drug interaction, *in vivo* pharmacokinetic properties, accurate therapeutics dose, possible routes of administration, and established convenient nanoformulation of sesamin.

Furthermore, sesamin's structure-activity relationship (SAR) should be determined to predict biological activities. Acquisition of more information about synergistic activities of sesamin in combination with others phytochemicals and existing drugs can increase activities of drugs with reverse the anticancer resistance pattern by modifying those existing drugs. In addition, sesamin can be analyzed in computational chemistry studies, i.e., docking analysis, neural networking study, and pharmacophore-based virtual screening campaigns for the drug discovery community. Successful performance of all approaches will make sesamin an effective chemotherapeutic anticarcinogenic agent in treating various subtypes of breast cancer.

## 9. Conclusion

**Figure 3:**
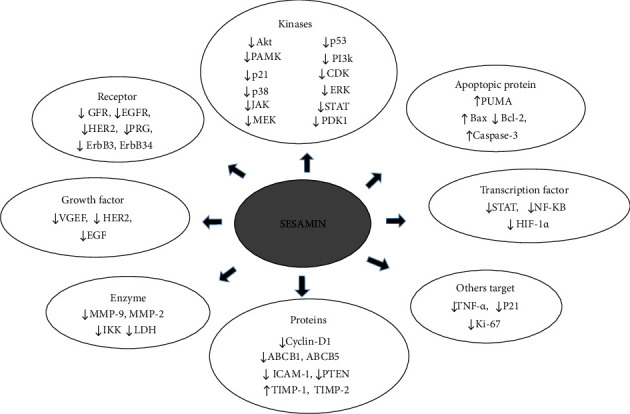
Overview of molecular targets influenced by sesamin in breast cancer. Studies have shown that sesamin can targets major molecular factors in breast cancer treatments. Downward directions (↓) represent downregulation, while upward directions (↑) represent upregulation.

Along with other plant species, *S. indicum*, the oilseed crop has nutritional, medicinal, and industrial significance and assists as the main plant source for acquiring large amounts of sesamin. This phytochemical possesses a crucial functional group that attributes several biological functions. The evidence with sesamin highlighted in this review is insufficient, but we provided a comprehensive overview of the potential anticancer mechanism against breast pathobiology in *in vitro and in vivo* studies. The regulation of PI3K/AKT, JAK/STAT, and MAPK signaling pathways has been linked to sesamin's tumor-inhibitory activities, mediated by some typical receptor estrogen, HER2, and EGFR. Similar to sesamin, some of the sesamin metabolites possess anticancer activities in numerous cancerous cell lines. The prominent anticancer mechanisms of sesamin regulate transcription factors, apoptotic proteins, enzymes, receptors, growth factors, cell cycle regulatory protein and enzymes, and other numerous targets (Figure 3).

**Figure 4:**
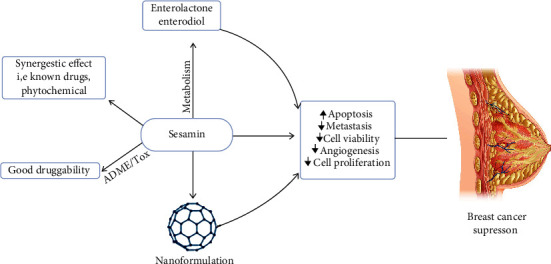
Summary of sesamin's pharmacological properties to pharmacological insight in breast cancer treatment.

Moreover, sesamin has been found to attribute additive or synergistic activity with other phytochemicals such as *γ*-tocotrienol, a type of vitamin E. Little study has addressed the contradictory effect of sesamin. Pharmacokinetics parameters stated that sesamin satisfied all common properties that indicate good candidates for future drug development to treat cancer patients and other several other diseases. However, in the future, more experimental studies need to be conducted (*in vitro*, *in vivo*, and clinical studies) to assess sesamin's efficacy on breast cancer and safety. Therefore, based on the review analysis (Figure 4), we hope that sesamin utilization in cancer biology can create an open door in the anticancer impact regarding breast cancer treatment as well as other diseases by using potential phytochemicals.

## Figures and Tables

**Table 1:** Reported plant sources of sesamin.

Name of the plant	Plant parts used	Ref.
*Sesamum indicum*	Leaves and seed	[28]
*Acanthopanax sessiliflorus*	Fruit	[37]
*Magnolia coco*	Stem	[32]
	Leaves	[33]
*Zanthoxylum tetraspermum*	Stem bark	[36]
*Zanthoxylum caudatum*	Stem bark	[36]
*Bridelia retusa*	Stem bark	[35]
*Zanthoxylum americanum*	Fresh roots and stems	[38]
*Glossostemon bruguieri (Desf.)*	Roots	[39]
*Piper longum*	Dried fruits	[40]
*Asarum heterotropoides* var. *mandshuricum*	Roots	[41]
*Fagara zanthoxyloides*	Roots	[42]
*Stemona collinsae*	Roots	[34]
*Cuscuta palaestina*	Entire plant	[43]
*Chrysanthemum cinerariaefolium*	Flower	[44]
*Flindersia pubescens*	Bark	[45]

**Table 2:** Summary of the mechanisms of action of sesamin in *in vitro* breast cancer models.

Sesamin/metabolites	Type of study *In vitro* and *in vivo*	Dose	Molecular mechanism	Molecular target	Ref
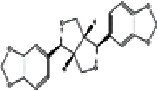 Sesamin	*In vitro* MDA-MB 232 and MCF-7	0-200 *μ*M	↓Cell proliferation ↓Migration	↓PDL-1 (both mRNA and protein) expression ↓P13K/AKT,NF-*κ*B, ERK, and JAK/stat signaling ↓MMP-9 and MMP-2 ↑Cell cycle arrest at the G1phase ↑PUMA and Bax	[69]
	*In vitro* MCF-7	0-100 *μ*M	↓Cell proliferation ↑Growth inhibition	↑G1 cell cycle arrest ↓Cyclin D1 protein ↑RB disphosphorylation	[73]
	*In vitro* MDA-MB 232 and MCF-7	51.1 *μ*M	↓Cell proliferation ↓Metastasis ↓Angiogenesis	↓Growth factor receptor ↓EGFR, HER2, and pMAPK expression ↓NF-*κ*B activation ↓I*κ*B*α* protein kinase ↓Bcl-2 and survivin ↓MMP-9, VGEF, and ICAM-1	[74]
	*In vitro* MDA-MB 232 and MCF-7	98.57 *μ*M	↑Cell cytotoxicity ↓Cell viability	Not mentioned	[76]
	I*n vitro* MCF-7	25-100 *μ*M	↓Cell proliferation	↓TNF-*α* and IKK	[82]
	*In vitro and in viv*o MCF-7, MDA-MB-231, and T-47D	0-50 *μ*M	↑Synergistic activity ↑Apoptosis	↓Overexpress of ABCB1 and ABCB5	[75]
	*In vitro* MCF-7	50 *μ*M	↓Cell viability ↑Apoptosis ↓Cell proliferation	↑G1 phase arrest, and CDK2 ↑Bax, caspase3, p^53^, and p^21^ ↑LDH release	[77]
	*In vitro* MDA-MB 232 and MCF-7	50 *μ*M	↓Angiogenesis ↓Macrophage-induced proangiogenic activity	↓Macrophage-induced VEGF ↓MMP-9 ↓ERK, JNK, PI3K, and NF-*κ*B ↓Cyclin D1 ↓Akt, HIF-1*α*, NF-*κ*B, and p38MAPK	[78]
	*In vitro* (MCF-7)	50 *μ*M	↓Cell growth ↓Cell viability	↑pS2 expression and progesterone receptor gene ↓ERE activation	[63]
	*In vitro* +SA mammary epithelial cell and MCF-7	60-120 *μ*M	↓Cell growth ↓Cell proliferation ↓Angiogenesis	↓EGF-induced ErbB3 ↓MEK1/2, ERK1/2, P13K, PDK1, Akt, p-NF-*κ*B, Jak1, Jak2, and Stat	[77]

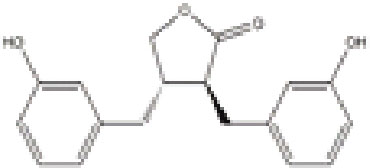 *Enterolactone*	*In vitro* MDA-MB-231	25-75 *μ*M	↑Anticancer activity ↓Migration	↓MMP-2 and MMP-9 ↑TIMP-1 and TIMP-2	[24]
	*In vitro* MDA-MB 231		↓Cell growth	↑S phage arrest ↓Cyclin E1, A2, B1, and B2 expressions	[80]
	*In vitro* MDA-MB-231	10 ng/ml	↑Anticancer mechanism	↓ERK/NF-*κ*B ↓MAPK-p38 ↓CD44 ↑E-cadherin, occluding	[81]

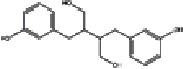 *Enterodiol*	*In vitro* MCF-7		↓Cell migration ↓Cell proliferation	↓MMP-2 and MMP-9	[72]

**Table 3:** Drug availability evaluation profile sesamin.

Category	Properties	Predictive remarks	Unit
Drug-likeness	Lipinski	Yes	Yes/no
	Veber	Yes	Yes/no
	Muegge	Yes	Yes/no
	Ghose	Yes	Yes/no
	Egan	Yes	Yes/no
	Bioavailability score	0.55	N/A

Absorption	Water solubility	-4.223	Log mol/l
	CaCO2 permeability	1.399	Log Papp (cm/s)
	Intestinal absorption(human)	97.81	% absorbed
	Skin permeability	-2.772	Log Kp
	P-glycoprotein substrate	No	Yes/no
	P-glycoprotein I inhibitor	Yes	Yes/no
	P-glycoprotein II inhibitor	No	Yes/no

Distribution	VDss (human)	-0.17	Log L/kg
	BBB permeability	-0.862	Log BB
	CNS permeability	-2.939	Log PS

Metabolism	CYP450 2C9 substrate	No	Yes/no
	CYP450 2D6 substrate	No	Yes/no
	CYP450 3A4 substrate	No	Yes/no
	CYP450 1A2 inhibitor	Yes	Yes/no
	CYP450 2C9 inhibitor	Yes	Yes/no
	CYP450 2D6 inhibitor	Yes	Yes/no
	CYP450 2C19 inhibitor	Yes	Yes/no
	CYP450 3A4 inhibitor	Yes	Yes/no

Excretion	Total clearance	-0.126	Log ml/min/kg

Toxicity	Skin sensitization	No	Yes/no
	Hepatotoxicity	No	Yes/no
	AMES toxicity	Yes	Yes/no
	hERG I inhibitors	No	Yes/no
	hERG II inhibitors	No	Yes/no
	T. pyriformis toxicity	0.34	Log *μ*g/l

Anticancer effect	P-GP inhibitor	Yes	Yes/no
	Aromatase	No	Yes/no
	ER binding	Yes	Yes/no
